# A Web-Based Telemanagement System for Patients With Complex Inflammatory Bowel Disease: Protocol for a Randomized Controlled Clinical Trial

**DOI:** 10.2196/resprot.9639

**Published:** 2018-12-21

**Authors:** Mariam Aguas, Javier del Hoyo, Raquel Faubel, Diana Muñoz, David Domínguez, Guillermo Bastida, Belén Navarro, Alejandra Barrios, Bernardo Valdivieso, Marisa Correcher, Pilar Nos

**Affiliations:** 1 Gastroenterology Department La Fe University and Polytechnic Hospital Valencia Spain; 2 CIBEREHD (Networked Biomedical Research Center for Hepatic and Digestive Diseases) Valencia Spain; 3 Health Research Institute La Fe Valencia Spain; 4 Physiotherapy Department University of Valencia Valencia Spain; 5 Joint Research Unit in Biomedical Engineering (eRPSS: IIS La Fe-UPV) Valencia Spain; 6 Connected Health Services Valencia Spain; 7 Home Care and Telemedicine Department La Fe University and Polytechnic Hospital Valencia Spain; 8 Red de Investigación en Servicios de Salud en Enfermedades Crónicas (REDISSEC) Madrid Spain; 9 Systems Department La Fe University and Polytechnic Hospital Valencia Spain

**Keywords:** inflammatory bowel disease, Crohn disease, ulcerative colitis, information technology, eHealth, telemedicine

## Abstract

**Background:**

Telemedicine has been successfully used to provide inflammatory bowel disease (IBD) patients with health care services remotely via the implementation of information and communications technology, which uses safe and feasible apps that have been well accepted by patients in remission. However, the design of telemedicine apps in this setting involves difficulties that hinder the adherence of patients to the follow-up plans and the efficacy of these systems to improve disease activity and quality of life.

**Objective:**

This study aimed to evaluate the development of a Web platform, Telemonitoring of Crohn Disease and Ulcerative Colitis (TECCU), for remote monitoring of patients with complex IBD and the design of a clinical trial involving IBD patients who received standard care (G_Control), nurse-assisted telephone care (G_NT), or care based on distance monitoring (G_TECCU).

**Methods:**

We describe the development of a remote monitoring system and the difficulties encountered in designing the platform. A 3-arm randomized controlled trial was designed to evaluate the effectiveness of this Web platform in disease management compared with G_NT and G_Control.

**Results:**

According to the schedules established for the medical treatment initiated (corticosteroids, immunosuppressants, or biological agents), a total of 63 patients (21 patients from each group) answered periodic questionnaires regarding disease activity, quality of life, therapeutic adherence, adverse effects, satisfaction, work productivity, and social activities. Blood and stool analyses (fecal calprotectin) were performed periodically. On the basis of the results of these tests in G_TECCU, alerts were generated in a Web platform with adapted action plans, including changes in medication and frequency of follow-up. The main issues found were the development of an easy-to-use Web platform, the selection of validated clinical scores and objective biomarkers for remote monitoring, and the design of a clinical trial to compare the 3 main follow-up methods evaluated to date in IBD.

**Conclusions:**

The development of a Web-based remote management program for safe and adequate control of IBD proved challenging. The results of this clinical trial will advance knowledge regarding the effectiveness of TECCU Web platform for improvement of disease activity, quality of life, and use of health care resources in complex IBD patients.

**Trial Registration:**

ClinicalTrials.gov NCT02943538; https://clinicaltrials.gov/ct2/show/NCT02943538 (Archived by WebCite at http://www.webcitation.org/6y4DQdmt8)

**International Registered Report Identifier (IRRID):**

RR1-10.2196/9639

## Introduction

### Background

Inflammatory bowel disease (IBD) comprises ulcerative colitis (UC) and Crohn disease (CD). The incidence of IBD is increasing gradually [1], and the prevalence of IBD in Spain is now 1 per 400 inhabitants [2]. Due to its chronic nature, IBD is associated with high levels of school and work disability, interference with social activities, and impairment of quality of life [3-6]. For these reasons, IBD generates a significant medical, social, and financial impact. Therefore, patients with IBD must undergo continuous and individualized monitoring to prevent structural damage and complications in the medium and long terms.

Changes in information and communications technology (ICT) over the last 10 years have affected the management of chronic diseases. Telemedicine is an ICT application that provides remote health care services, that is, without the need for direct contact with the patient. It has been associated with a high degree of satisfaction in patients with chronic diseases such as chronic obstructive pulmonary disease, diabetes mellitus, and congestive heart disease [7-9].

Telemedicine apps have been used to improve adherence to treatment and patient education in IBD (teletraining) [10]. Several studies have shown these apps to be feasible and acceptable for patients with IBD, and the level of satisfaction with them is at least comparable with that of patients who receive standard face-to-face care [11-14]. In addition to teletraining systems, Web-based apps have been developed to provide educational support to patients, thus potentially giving them increased independence for starting or maintaining treatment during a flare-up and for acquiring knowledge about the disease. However, these findings have not been consistent across different populations [15,16].

Current evidence suggests that the use of ICT is a safe way of monitoring patients with IBD, mainly UC in remission or with mild activity [14,16-18]. However, in some studies carried out to date, telemedicine apps require home installation and eventual repairs [14] with differences in reproducibility according to the population in which they are applied [16]. These hindrances interfere with the patients’ adherence to follow-up plans and the efficacy of these systems to improve disease activity and quality of life. Moreover, it remains necessary to perform studies to confirm these findings in patients with more complex disease who require treatment with immunosuppressive or biologic drugs and to analyze the impact of telemedicine on the cost of care.

### Study Objective

Therefore, this study addresses the main questions related with the use of a Web telemonitoring program in patients with complex IBD, a difficult context in which telemedicine has not been previously used and requires a closer follow-up. We describe the development of the remote monitoring platform *Telemonitorización de la Enfermedad de Crohn y Colitis Ulcerosa* *(Telemonitoring of Crohn Disease and Ulcerative Colitis,* TECCU) and the clinical trial protocol for evaluation of the intervention compared with nurse-assisted telephone care (G_NT) and standard care (G_Control). Moreover, we detail the selection of indices and questionnaires used to measure the study outcomes, the patient population, and the difficulties found in the design and implementation of the trial, with the aim of helping other investigators in the development of telemonitoring interventions in the setting of complex IBD.

## Methods

### Study Design

**Figure 1:**
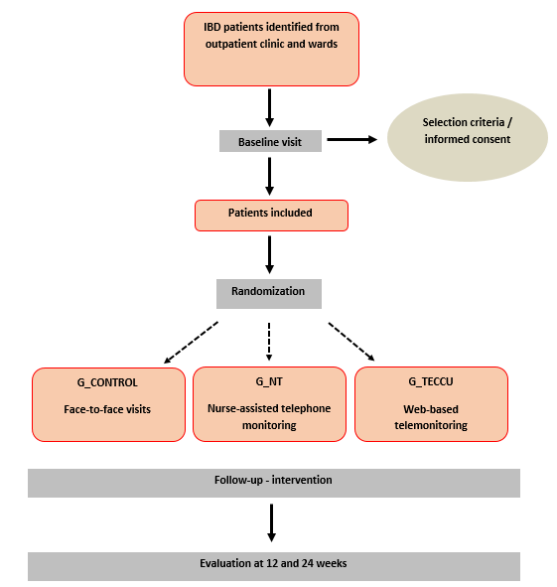
Flowchart of study participants. IBD: inflammatory bowel disease; G_Control: standard care; G_NT: nurse-assisted telephone care; G_TECCU: care based on distance monitoring.

We developed a Web-based telemanagement system—TECCU—for patients with complex IBD. To evaluate the effectiveness of the Web app for control of disease activity, we designed a randomized controlled clinical trial comparing follow-up of patients through the Web-based app (G_TECCU) with G_NT and G_Control. Eligible patients were randomized to 1 of the 3 groups in a 1:1:1 ratio using a Web-based tool to generate the random allocation sequence and ensure allocation concealment. All patients attended study visits at baseline and at 12 and 24 weeks, in addition to routine visits to the IBD clinic or telephone consultations based on group assignment at randomization (Figure 1). Disease activity, health-related quality of life (HRQOL), adverse effects, adherence, and use of health care resources were measured at baseline and during the 24-week follow-up. In CD patients, disease activity was measured with the Harvey-Bradshaw index (HBI), and in UC patients, it was measured with the simple clinical colitis activity index (SCCAI, also known as the Walmsley index) for remote checkups and the 9-point Mayo score for face-to-face visits.

### Patient Selection

Patients were recruited from the IBD unit, La Fe University and Polytechnic Hospital (tertiary referral center), Valencia, Spain. The reference population was 320,000 inhabitants in 2016, although the IBD unit was available to patients from the whole Autonomous Community of Valencia. Thus, the potential population comprised 640,000 inhabitants. The hospital serves more than 1500 patients with IBD (over 400 of them treated with biological agents), has 2 specialist IBD nurses, and provides an email and telephone consultation structure for IBD patients to contact the hospital.

The study participants had been diagnosed with IBD at least 6 months previously according to internationally accepted criteria [19,20] and had to meet all the following inclusion criteria: age ≥18 years, CD or UC with moderate or severe activity/initiation of therapy with immunosuppressive or biological agents, and written informed consent to participate in the study. Patients were excluded if they met any of the following criteria: inability to speak or read Spanish; suspicion that monitoring will not be completed (outpatients); inability to manage a mobile phone/tablet or internet or not having a telephone line; uncontrolled medical or psychiatric disease, pregnancy, active perianal disease, presence of ileorectal or ileal pouch-anal anastomosis; receipt of definitive ileostomy; and participation in another experimental study during patient enrollment.

To evaluate differences in the percentage of patients in remission and changes in the score of activity indices between the 3 groups, we calculated that a sample size of 60 patients (30 CD patients and 30 UC patients) was necessary. During the study period, patients were included consecutively from the outpatient clinic of the IBD unit or the gastroenterology department if they had been admitted for a flare-up. A total of 68 IBD patients who met selection criteria were invited to participate in the study. Of these, 4% (3/68) declined to participate owing to inaccessibility to the internet at home and 3% (2/68) did not meet the inclusion criteria. The remaining 63 eligible patients provided their informed consent and were randomly assigned to G_TECCU, G_NT, and G_control (21 patients in each group). All patients enrolled were included in a likelihood-based analysis.

### Recruitment and Random Assignment

Patients were included consecutively from the outpatient clinic of the IBD unit or the gastroenterology department if they had been admitted for a flare-up of IBD. During the visit, the inclusion and exclusion criteria were verified and the patient was informed about the study through the patient information sheet. If the patient agreed to participate, he or she signed the informed consent document. Patients were assigned to the different groups using a secure and independent randomization tool. Once a number was assigned, it could not be reassigned.

Eligible patients were assigned to the groups through a computer platform that ensured that the researchers could not modify a patient’s group. This process was performed using a block randomization method between the groups (1:1:1) with concealed allocation of participants. The blocks were numbered and ordered using a randomly generated sequence. Members of the research team who were in contact with patients did not have access to the randomization tables or lists.

### Interventions

#### Types of Interventions

The trial had 3 arms with 3 different interventions (Figure 1):

1. G_control: Patients in the control group received the usual care provided in the IBD unit (outpatient clinic) to patients with moderately to highly complex IBD. We also provided on paper all educational material about IBD available for the remote monitoring patients as well as information on prevention and written action plans in case of flare-up to make the 3 groups more comparable. Moreover, the clinical status of patients was recorded weekly during the first 12 weeks and subsequently every 2 weeks until the end of the follow-up in a diary designed on paper. This care was complemented by ad hoc hospital care in the case of flare-ups or if patients’ health deteriorated for any reason. Ad hoc intensive care was maintained until patients’ condition stabilized, at which point he or she returned to follow-up based on standard care in the unit. The intervention was limited to recording of baseline variables, follow-up for identification of end points, and administration of activity indices and questionnaires on HRQOL, satisfaction, and work productivity.
2. G_NT: Patients in this group were asked about their health through a telephone call from nursing staff in the IBD unit, and we provided these patients with all educational elements and action plans available in the other 2 groups. Telephone assessment was performed periodically using structured interviews to evaluate clinical status with intensified schedules, in a similar manner to the G_control. The actions carried out depended on the results of the interview and the intervention protocol.
3. G_TECCU: Follow-up and monitoring in this group were performed telematically using the integrated platform for management of chronic patients NOMHADchronic, which was set up to meet the specific needs of IBD patients. Patients connected to the platform via the internet using a computer or an app on a mobile phone or tablet and had to complete questionnaires (both scheduled questionnaires and those requested by the patient at other time points) and introduce relevant follow-up data (eg, vital signs and analytical data). They also received advice, reminders, educational material about their disease, and information on prevention. Case managers received the information from each patient, which was filtered using an intelligent prioritization system and generation of alerts and push notifications according to an integrated intervention protocol. Physicians from the IBD unit were also able to access individual patients’ data and received referrals about alerts and push notifications that had to be managed by medical staff.

#### NOMHADchronic Platform

The NOMHADchronic platform is an innovative technological system that was designed to boost the rollout of services for the management of chronically ill patients. The platform covers the needs of patients, professionals, and the organization managing the care process and coordinates the work of the professionals involved. This efficiency-based multiplatform system provides a flexible, integrated solution that can be adapted to provide various services. Artificial intelligence enables it to provide support for decision making. It was developed following the Health Level 7 (HL7) version 3 standard, thus enabling its easy integration with the electronic clinical history. It can be set up in such a way that plans and thresholds can be personalized according to each patient’s profile. The system also makes it possible to create combined alerts using several variables, thus facilitating a holistic approach to the patient and not only to the specific diseases diagnosed.

This program was initially designed to manage patients with other chronic diseases such as cardiovascular diseases or diabetes mellitus. The telemanagement care system was developed in collaboration with La Fe telemedicine unit, Tecnología Salud y Bienestar SA, and patients from our IBD unit. With the aim of adapting the platform to the particular needs of complex IBD patients, we performed consecutive meetings with patients and researchers to modify the original modules of NOMHADchronic. We changed the elements of the platform to monitor disease activity and other clinical outcomes with specific indices, the normal values, and parameters recorded in the blood tests and fecal calprotectin as well as the follow-up schedule according to clinical guidelines in the management of IBD patients. Moreover, we incorporated educational elements to improve disease knowledge of patients and empowerment through interactive materials.

**Figure 2:**
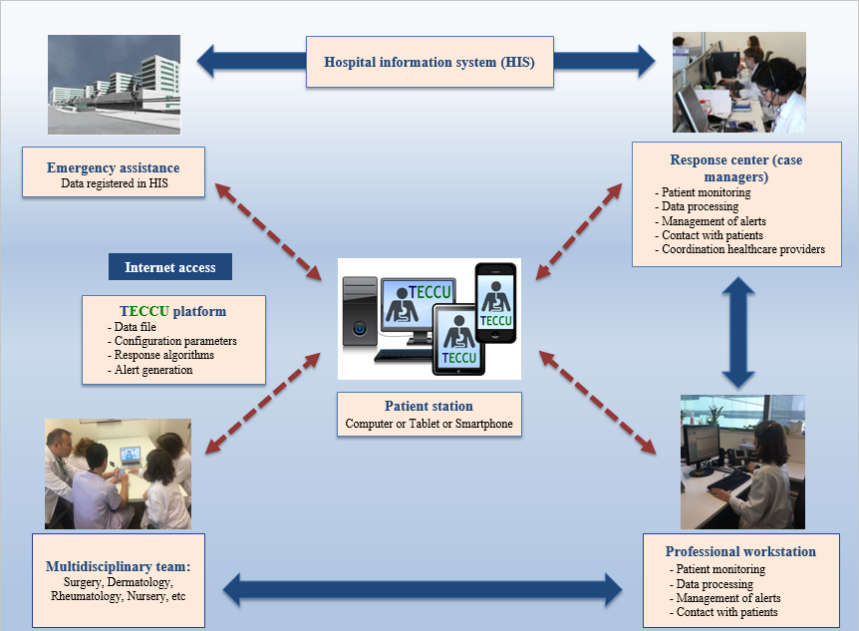
Telemonitoring system for patients with complex inflammatory bowel disease. TECCU: Telemonitoring of Crohn Disease and Ulcerative Colitis.

NOMHADchronic comprises NOMHADcore (the nucleus of the platform), the workstation (for health professionals), the response center (case managers), and the patient station (on the Web platform, tablet, or mobile phone). The solution involves various modules to cover all aspects of management of chronically ill patients: clinical information relative to patients’ status, support for coordination of the different health care providers, logistics management, human resources management, and integration with monitoring devices and accessories (Figure 2). The tool concentrates on 4 main functional areas: innovative education, multimodal care communication, monitoring, and personal clinical history.

Researchers designed the follow-up protocol according to national and European guidelines [19-21] and set up the platform to include the components described in Textbox 1.

**Components of the development of the telemonitoring of Crohn disease and ulcerative colitis (TECCU) platform.**

Components of the development of the telemonitoring of Crohn disease and ulcerative colitis (TECCU) platform:

- Detailed monitoring plan
- Definition of the main indicators and alarms
- Specific interventions for the patient
- Specific interventions for health professionals
- Configuration of the user environment in the health professional’s app
- Configuration of the user environment in the patient’s app

**Figure 3:**
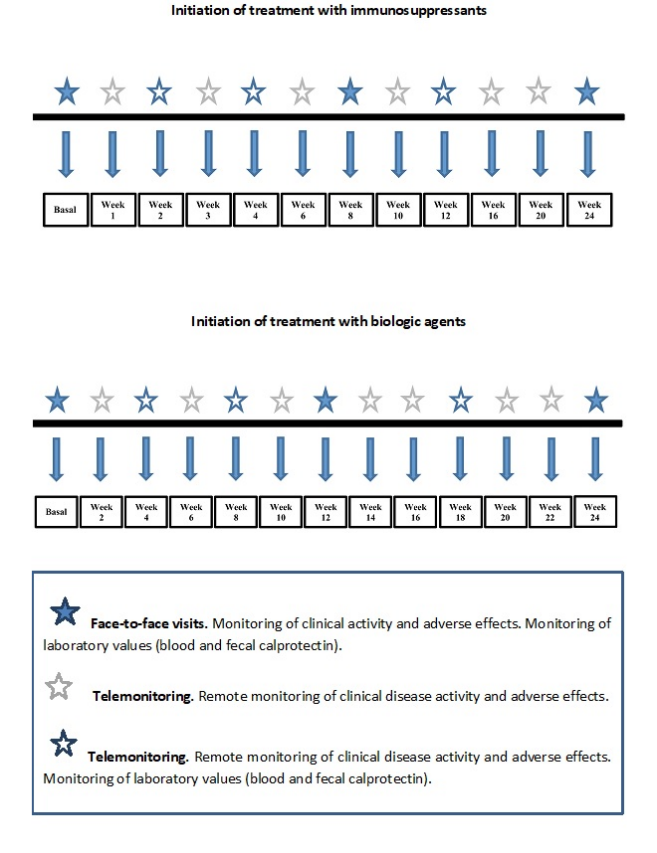
Follow-up timeline for G_TECCU receiving treatment with immunosuppressants or biologic agents.

#### Follow-Up Schedule

Irrespective of the study arm, the patients included followed a periodic monitoring program adapted to the type of medication they were taking (corticosteroids, immunosuppressantsor biologics). In the case of combined treatment with immunosuppressants and biologics, the follow-up program was similar to that of patients who were taking immunosuppressants alone. Furthermore, patients from the 3 arms who took the same type of drug underwent identical monitoring, which was performed along a time line for patients treated with immunosuppressive drugs and another for patients treated with biologics (Figure 3). The difference lay in the fact that patients from G_TECCU were monitored via the NOMHADchronic system, G_NT via a telephone line managed by nursing staff from the IBD unit, and G_control with the usual face-to-face visits. Irrespective of the group assigned and treatment prescribed, all patients had to visit the IBD unit at baseline, 12 weeks, and 24 weeks.

#### Telemonitoring of Crohn Disease and Ulcerative Colitis Self-Testing

**Figure 4:**
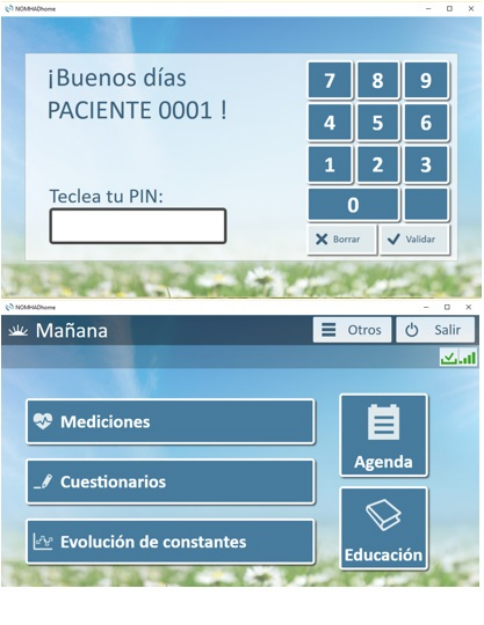
Home page of the NOMHADhome platform, patient version. Translations for the screenshot: Mañana: Morning; Mediciones: Statistics; Cuestionarios: Questionnaires; Evolución de constants: Vital signs; Educación: Education; Buenos días paciente 0001: Good morning patient number 0001; Teclea tu PIN: Please type your PIN number; Otros: Other; Salir: Disconnect.

**Figure 5:**
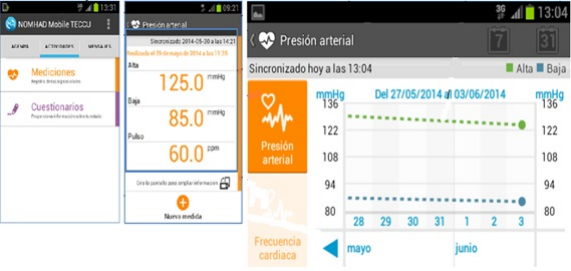
Home page of the NOMHADmobile platform and access to vital signs, patient version.

Patients monitored via the Web-based system used NOMHADhome, which was installed from a page that was supplied to the patients and could be accessed via the internet. The resources of the NOMHADhome platform were also available on the NOMHADmobile app, which patients could download onto their mobile phone. After inclusion, these patients automatically received an email with a personal access code that enabled them to access the NOMHADhome platform. Each patient profile contained the following data: contact information (address, email, and phone number); active IBD medications; testing schedule (blood and stool tests); log of disease activity, medication use, body weight, vital signs, and quality of life; alerts and action plans; progress of inflammatory activity and vital signs in the form of graphs; electronic messaging to the study nurse coordinator and the health care provider; and educational tips (Figures 4 and 5).

Formative sessions of 30 to 60 min were performed with patients and investigators to improve practical utilization of the platform before starting follow-up. During telemonitoring, patients accessed via the internet the Web platform with their personal password and responded to different aspects of their disease. In all checkups, they introduced their vital signs, IBD symptoms to measure disease activity, medication adherence, and adverse effects to medication. In the main menu of the platform, patients accessed the questionnaires by clicking on the specific icons designed for this purpose and could then answer the multiple choice and yes or no questions needed to complete these items. When it was necessary to fulfill the results of blood tests or fecal calprotectin performed in their medical center, patients could introduce their results in different text boxes for each parameter similarly. Thus, the patient was able to self-complete all the clinical and analytical variables necessary to evaluate his or her disease status at each of the checkups programmed in their follow-up time line. In addition to these fixed monitoring points, the patient could complete the questionnaires voluntarily if his or her clinical status deteriorated.

To assess disease activity, CD patients calculated their modified HBI via text messaging. The HBI is a validated activity index that has been shown to correlate well with the criteria of the standard instrument for assessing disease activity for CD, the CD activity index [22]. The HBI includes 5 clinical variables that apply to the 24 hours since the previous evaluation on overall well-being, abdominal pain, number of liquid stools per day, presence of abdominal mass, and complications. For UC, patients completed the SCCAI via text messaging. The SCCAI has been shown to correlate well with other disease activity indices for UC and was recently validated for self-administration via a Web-based tool [23,24]. The SCCAI contains 6 questions on number of daytime and nocturnal bowel movements, urgency, blood in stool, general well-being, and extraintestinal manifestations of disease. In addition to disease activity, during clinical controls, patients answered a series of questions about medication adherence and adverse effects.

After completion of a scheduled or voluntary monitoring questionnaire, a personalized action plan was designed depending on the results obtained. On the basis of the responses to the different outcomes evaluated, the platform stratified patients in the professional workstation according to the severity of their health status in different levels of severity. Health care providers were then able to respond to patient needs according to each health status. The platform proposed a specific intervention according to preestablished algorithms and medical providers, in coordination with case managers, and finally implemented a customized action plan for each case as described in the following section.

Educational materials were also included in a specific section available in the main menu of the platform. At any time, patients could review the information adapted by our research group and they could answer different questions related to the information provided to check their IBD knowledge. Moreover, the platform allowed the interaction with health care providers through a messaging menu to contact case managers and medical providers as needed. Patients could ask questions related to their disease evolution or treatment by texting a message with the keyboard. These messages were frequently reviewed so that they were answered by our medical or nurse staff after 24 to 48 hours.

#### Description of Telemonitoring of Crohn Disease and Ulcerative Colitis Alerts and Action Plans

We established individualized alerts and action plans based on the answers to questions about the activity index, adverse effects, and results of blood and stool parameters. A scale of values was assigned for each alert depending on severity (green, yellow, orange, and red zone). For example, according to the activity index score (remission or mild, moderate, or severe disease), the patient is categorized in the green, yellow, orange, or red zone, and the health professional can select 1 or several actions to immediately begin self-testing (Tables 1 and 2). Each level of alert was associated with a preestablished intervention plan that involved closer monitoring, changes in drug dose, or even referral to the hospital emergency service or an outpatient clinic. These interventions were managed by nursing or medical staff depending on the degree of severity of the alert. Once the disease was in remission again (green zone), the patient had to continue with the initially programmed follow-up. In some cases, the criteria for entering each zone could be modified by the researcher to adjust the specific clinical status of each patient.

**Table 1:** Action plans according to Harvey-Bradshaw index activity index scores.

Harvey-Bradshaw index	Action plan
Score >16: Severe activity^a^	Intervention 1: Send patient to hospital immediately^a^
Score 8-16: Moderate activity^b^	Intervention 1: Request priority appointment in outpatient clinic^b^ Intervention 2: Adjust immunosuppressants and biologics^b^
Score 5-7: Mild activity^c^	Intervention 1: Monitor 2-3 times per week^c^ Intervention 2: According to new score Intervention 3: Adjust new medication or add new treatment
Score <5: Disease in remission^d^	Continue with monitoring program^d^

^a^Red zone.

^b^Orange zone.

^c^Yellow zone.

^d^Green zone.

**Table 2:** Action plans according to Simple Clinical Colitis Activity Index (SCCAI) activity index scores.

Simple Clinical Colitis Activity Index (SCCAI)	Action plan
Score >5^a^	Contact the physician^a^
Score 3-5^b^	Follow-up with repeat of SCCAI in 5 days^b^
Score ≤2^c^	Continue with the same monitoring program^c^

^a^Red zone.

^b^Yellow zone.

^c^Green zone.

To ensure that patients in the G_TECCU arm adhered to the requirements of remote monitoring, 2 days before each monitoring date, they received a message via email if they were using the NOMHADhome app or a message via their mobile phone if they were using NOMHADmobile. Similarly, the patients were sent a reminder if they did not enter their data on the scheduled date.

Furthermore, all patients had access to tools that taught them about their disease. In G_TECCU, information was available on the NOMHADhome platform itself; in the G_Control and G_NT arms, the patients received written documents with the same information.

#### Health Data Security

TECCU Web platform protects the confidentiality of health data. The access to patient station and to workstation requires a personal password only known by the patient and health care providers, respectively. Moreover, health care providers register patients in the platform with a generic name and a code only identifiable by investigators. Finally, to avoid data correlation by a nonauthorized person, data included in the Web platform are not connected to other hospital information systems. Thus, only case managers and health professionals can see all the clinical history separately.

#### Notification of Adverse Effects

TECCU is a minimal risk system. The characteristics of the interventions in this study mean that patients are not expected to experience direct adverse effects. The interventions were proposed to control and achieve longer remission periods in patients with IBD. Therefore, they cannot cause lesions or damage the patients’ health, since we did not test a new experimental drug; on the contrary, we used a Web-based telemonitoring system to give indications when flare-ups are detected or when these become more severe. With respect to the platform, a risk minimization study was performed to ensure the accomplishment of ethical norms and that the appropriate ethics committees approved the study. In any case, we recorded all adverse events occurring from the moment the patient gave his or her consent to participate in the study until 28 days after the study finished.

### Statistical Analysis

#### Sample Size Calculation

It was decided that the most efficient means of determining differences between the 3 groups (G_control, G_NT, and G_TECCU) was by contrasting differences in activity indices for the diseases treated (CD and UC). Given that the scales differ, the analysis was stratified by performing a comparison for patients with UC and another comparison for patients with CD. The sample size was calculated by estimating that to detect a difference of 3 points in the HBI, taking into account an SD in the index of 4, a power of 80%, and an alpha significance level of .05, a total of 30 patients with CD (10 per arm) would be needed. Moreover, for a difference in the Mayo index of 2 points, taking into account an SD of 2.5, a power of 80%, and an alpha significance level of .05, a total of 30 patients with UC (10 per arm) would be needed. Therefore, the overall sample size was 60 patients (20 per arm).

We also stratified patients globally (CD and UC) by comparing those in remission or with inflammatory activity, irrespective of severity (mild, moderate, or severe).

#### Statistical Analysis Plan

First, we described the characteristics of patients in the test and control groups (by applying appropriate estimators according to the type of variables with their respective CIs for means or proportions) and evaluated possible differences between the groups using tests to assess differences in means or proportion. Second, we evaluated differences in the main outcome measure and in the secondary outcomes, again using tests to assess differences in means and proportions, as applicable. We then used linear regression models (or logistic regression models) to evaluate the significance and magnitude of the effect of the intervention while controlling for possible differences in the characteristics of patients from both groups. We generally used backward-forward models by initially constructing complete models and excluding nonsignificant variables one by one (*P* for removing variables=.10), although we tried to include the previously removed variables after each exclusion (*P* for including variables=.05). The yield of the models was evaluated using the coefficient of determination and, in the case of logistic regression, by evaluating its discriminative capacity (C-statistic) and calibration (Hosmer-Lemeshow test). The statistical analyses were performed using R version 3.5.1 (R Foundation for Statistical Computing, Vienna, Austria). The analysis was performed on an intention-to-treat basis.

Costs were evaluated from a social perspective including direct health care costs. The unit cost was obtained by measuring the consumption of resources and applying the official price list of the Valencian Health Agency or other official bodies. When these were not available, they were estimated by evaluating the time and cost of the resources involved.

### Ethical Considerations

The study was carried out in accordance with the Declaration of Helsinki on ethical principles for medical research involving human subjects, as adopted by the General Assembly of the World Medical Association (1996). We followed the protocol, the principles of Good Clinical Practice, the guidelines of the International Conference on Harmonization, and the official regulations of the participating centers. The study protocol was reviewed and approved by the local independent ethics committee of La Fe University and Polytechnic Hospital, Valencia, Spain , the regional independent ethics committee (Comité Ético Autonómico de Estudios Clínicos de Medicamentos y Productos Sanitarios de la Comunitat Valenciana; CAEC), and the Spanish Agency of Medicines and Medical Devices (Agencia Española de Medicamentos y Productos Sanitarios; AEMPS). The study was carried out by persons with the appropriate scientific and medical qualifications. The benefits of the study were proportional to the risks. The rights and well-being of the participants were respected at all times. According to the physicians involved in the study, the risks did not outweigh the potential benefits, and each participant provided his or her informed consent without coercion. The trial was registered at clinicaltrials.gov with the identifier NCT02943538. The results will be published according to the CONSORT guidelines.

### Study Outcomes

Participants attended study visits at baseline, 12 weeks, and 24 weeks, in addition to routine visits scheduled for their clinical care. The variables measured at baseline were sociodemographic variables, disease activity, HRQOL, adverse events, adherence, and patient satisfaction. The results were recorded on a paper case report form designed ad hoc for the study. The same approach was used at 12 and 24 weeks. In addition, the hospital information system was consulted to collect data on mortality and variables associated with the consumption of hospital resources (emergency and scheduled visits to the outpatient clinic, visits to the emergency department, emergency and scheduled hospital admissions during follow-up, and surgical procedures). This information was used in the cost-effectiveness study, which was based on direct health care costs.

#### Primary End Point

The primary objective of the study was to evaluate clinical remission at 24 weeks. In addition, clinical disease activity was evaluated at each of the checkups scheduled in the patients’ time line. Remission was evaluated using the HBI for patients with CD [22]. In the case of patients with UC, we used the SCCAI [23] for remote checkups, together with the partial Mayo score for face-to-face visits.

Patients with CD and an HBI <5 were considered to be in clinical remission, whereas patients with scores of 5 to 7, 8 to 16, or >16 were classed, respectively, as having mild, moderate, or severe activity [22]. For remote checkups in patients with UC, clinical remission was defined as an SCCAI ≤2, whereas values of 3 to 5 or >5 points were classed as mild-to-moderate or severe activity, respectively [25]. In the face-to-face visits, clinical remission was defined as a partial Mayo score ≤2 and no individual Mayo subscore >1; scores of 2 to 5, 6 to 8, and 9 points were defined, respectively, as mild, moderate, and severe disease activity [26].

Laboratory parameters were measured at baseline and at each subsequent visit according to the individual patient’s schedule and included complete blood analysis with nutritional profile and C-reactive protein (mg/L) and fecal calprotectin (µg/g) to assess inflammatory activity. The results obtained could lead to changes in the medication prescribed. The alert generated led to an intervention. Fecal calprotectin was assessed at baseline and 12 and 24 weeks after inclusion, although their values did not lead to a specific intervention.

#### Secondary End Points

##### Health-Related Quality of Life and Impact on Activities of Daily Living

The HRQOL of the patients at inclusion and at week 24 or at the end of the follow-up visits was evaluated using the following 2 tools: a specific questionnaire, the IBD Questionnaire 9 (IBDQ-9), and a generic questionnaire, EuroQol-5D (EQ-5D). IBDQ-9 has been validated for IBD and correlated extremely well with clinical activity [27]. The Spanish version of IBDQ-9 has been validated and correlated well with the IBDQ-36 questionnaire in Spanish [28]. EQ-5D is a generic questionnaire that has been used in patients with various chronic diseases, such as IBD, and has been validated in Spanish [29]. This instrument provides a global value for HRQOL and a visual analog scale (VAS). It is known that responses to EQ-5D items and the VAS score are better for patients in remission than for patients with active disease [30].

Furthermore, the impact of disease on work productivity and activities of daily living was evaluated using the Work Productivity and Activity Impairment questionnaire, which patients completed at baseline and at week 24. The questionnaire comprises 6 questions on the effect of the disease on work and activities of daily living during the previous 7 days. The greater the score on the questionnaire, the greater the effect is on work and daily activities. The Spanish version has been validated and has been shown to be reproducible in patients with CD [31].

##### Evaluation of Adherence and Adverse Effects

Adherence was evaluated using the Morisky-Green index [32], which has been used in clinical trials to evaluate adherence in patients with IBD [14]. We consider adherence to be adequate when the patient responds to all questions correctly and inadequate if any answer was associated with nonadherence.

Furthermore, patients responded to a series of questions related to the onset of specific adverse effects to the drugs they were taking at the time. These checkups reflect the adverse effects of immunosuppressive or biologic agents, as preestablished by the research team in the NOMHADchronic app.

##### Assessment of Utilization of Health Care Resources

We recorded information from each patient on the use of health care resources, specifically the number of unscheduled visits to the outpatient clinic, visits to the emergency department, and nonscheduled admissions to hospital. This information was recorded using the hospital information system, Orion Clinic, which has been certified by the Health Information and Management Systems Society and is used in daily clinical practice in La Fe University Hospital. We also recorded the following: the reason for the visit to the outpatient clinic, visit to the emergency department, or hospital admission; whether or not IBD-related surgery was necessary; and the total length of hospital stay. In addition, we recorded all deaths during the study (whether related to IBD or not).

##### Assessment of Satisfaction With Care

Patient satisfaction with the care received was evaluated at 24 weeks using an adapted version of the Client Satisfaction Questionnaire, which comprises 6 questions on the quality, usefulness, and viability of the care received as a result the intervention [33].

## Results

This project was funded in 2012, and the platform was installed and configured from 2013 to 2014. Enrollment started in October 2014 and it finished in June 2016. At the time of this protocol’s submission, data analysis was underway, and the first results are expected to be published in 2018.

## Discussion

### Importance of the Study Results and Principal Findings

Efforts, to date, have focused on telemonitoring systems in an attempt to positively influence adherence to treatment and knowledge of the disease in the follow-up of patients in remission or with mild-to-moderate disease [14,16]. This study is the first randomized controlled clinical trial to evaluate the efficacy of a remote Web-based management program for the follow-up of patients with IBD of moderate-to-high complexity. We compared standard face-to-face or nurse-assisted telephone follow-up with remote monitoring based on a Web app (NOMHADchronic) for control of inflammatory activity, quality of life, adverse effects, adherence, and use of health care resources over a 24-week period. Patients with moderate-to-high disease complexity are considered to be those who start treatment with systemic corticosteroids, immunosuppressants, or biologics for control of inflammatory activity.

There is still little evidence on the role of telemedicine in monitoring health outcomes and reducing health care costs in the IBD setting. In 2001, Robinson et al [17] successfully implemented a self-care plan in patients with UC in remission. A total of 203 patients were randomized to G_Control or to self-management. During the 1-year study period, relapses in the self-management group were treated earlier and were shorter in duration, and utilization of health care resources decreased. Over a decade later, Cross et al [14] used a remote control system, Home Automated Telemanagement (HAT), to provide a secure patient portal to monitor symptoms, generate alerts, and inform patients about the disease in a randomized clinical trial. However, in this study, no significant differences were noted in disease activity and quality of life between patients followed with the HAT system and usual care, probably due to the small sample size and the design of the platform, which required home installation and repairs. To avoid these problems, the same group designed afterward a Web telemanagement program via mobile phone [34].

Web-based programs are a new and easy-to-use approach in telemanagement. The implementation of such programs reduces the costs of telemanagement because home installation is not required. Elkjaer et al [16] performed a pioneering study in Web-based management in which the concept of *constant care* was introduced for patients with UC. A randomized controlled trial comparing Web-based monitoring and standard follow-up was carried out with 333 patients with UC treated with 5-aminosalicylic acids (5-ASA) at hospitals in Denmark and Ireland. The remote care method was safe and feasible. The authors observed a trend toward higher adherence in patients managed using a Web-based system and that the duration of relapses was shorter than that in the G_control. These findings were associated with the ability of the Web-based program to empower patients to self-initiate 5-ASA in case of flare-ups. Moreover, in the experimental arm, patients made more phone calls and sent more emails but attended fewer face-to-face visits, and cost savings per patient-year were recorded.

Given that telemonitoring apps are well accepted by patients and seem to be a safe approach for follow-up of patients with less complex IBD (in remission or with mild activity), we designed a controlled, 3-arm clinical trial to compare the effectiveness of G_TECCU for the monitoring of inflammatory activity in patients with IBD of moderate-to-high complexity with G_control and G_NT. Our study enabled us to compare the main strategies applied to date in the follow-up of patients with IBD (standard or telephone support by a nurse) with remote monitoring. In particular, an easy-to-use Web-based program with a Web app (NOMHADchronic) would likely improve health outcomes and reduce costs more than other systems for remote management of the disease (eg, those that could require reparations at home) [35].

Another critical issue was the selection of activity index and biological tools for measuring disease activity and secondary outcomes remotely. We used clinical activity indices (HBI, SCCAI, and partial Mayo score) to evaluate the primary objective of the study. The SCCAI was previously used to evaluate the ability to control disease via telemonitoring systems [16] and was shown to correlate well with other, more complex activity indices that require endoscopy [36]. Similarly, the reduced 6- and 9-point versions of the Mayo index, which do not require endoscopy, have proven to be capable of detecting a clinical response to treatment, as is the case with the full Mayo score [26]. The partial Mayo score is only used at face-to-face visits because one of its items must be assessed by a physician. Nevertheless, as clinical indices may not reflect the true inflammatory activity of the disease, biological markers (C-reactive protein and fecal calprotectin) were used for the global evaluation and comparison of the 3 groups at the baseline and follow-up visits, which are sensitive enough to detect mucosal inflammation [37].

### Strengths and Limitations

Regarding the development of the TECCU Web program, the main difficulties were related to the design of an easy-to-use system, which is compatible with a broad group of devices. We designed an interactive tool, which allows patients to register their evolution at each moment, through a simple and structured main menu containing all relevant aspects to control IBD activity. A practical problem with the use of TECCU was its incompatibility with Microsoft Internet Explorer Web browser, but patients and researchers were able to open the TECCU platform using Mozilla Firefox or Google Chrome, and there was a version available for Windows and Mac operating system computers and Android or iOS mobile phones. Another issue was the absence of videoconference, but the electronic messaging and the intelligent alert plans included in the platform allowed coordination of health care providers to provide response to medical problems after 24 to 48 hours. Our study is subject to a series of limitations. First, we included patients with IBD managed at a tertiary referral hospital, and the exclusion of patients with suspicion that monitoring will not be completed is subject to difficulties in determining the ability and willingness to use ICT in each case, which represent a potential source of bias. According to guidelines for management of IBD, patients evaluated at referral centers often have more complex disease, are often more difficult to treat, and need aggressive therapies. Patients treated in community hospitals, on the other hand, generally have milder disease and require less aggressive treatment. However, the NOMHADchronic program may also help community physicians to be more aware of the guidelines, and it could even be more effective in patients who can self-manage with less aggressive treatments, which may be easier to implement. Second, we did not perform colonoscopy in all patients because it would increase the costs of the clinical trial, and colonoscopy is not performed routinely in all patients in clinical practice. Nevertheless, we intend to analyze the subgroup of patients who underwent routine colonoscopy. Third, due to the kind of interventions assessed, neither the patients nor the researchers were blinded with respect to the intervention, but the results were analyzed by an independent statistician who was blinded to group identification.

### Conclusions

In conclusion, the development of a Web-based program for the management of patients with IBD proved challenging in terms of providing adequate remote monitoring of the disease and acceptance of the system, mainly as a result of including patients with moderate-to-severe disease. We describe the difficulties we faced during the design of a randomized clinical trial to compare Web-based monitoring of patients with standard face-to-face monitoring and telephone monitoring. We aimed to aid other investigators in the development of telemonitoring interventions and the selection of methods to measure health outcomes remotely as well as the groups of patients who benefit from this approach during the design of future studies in the IBD setting. The results of our trial will show the impact of the TECCU telemonitoring system on disease activity, quality of life, and health care costs through continuous care and patient empowerment. Future studies are necessary to validate the NOMHADchronic remote monitoring program in other, specific groups of patients, namely, those with noncomplex disease, those with limited access to medical care or social support, and patients who may not be able to attend a clinic with specialized IBD care.

## Abbreviations

CD: Crohn disease
EQ-5D: EuroQol-5D
G_Control: standard care
G_NT: nurse-assisted telephone care
G_TECCU: care based on distance monitoring
HAT: Home Automated Telemanagement
HBI: Harvey-Bradshaw index
HRQOL: health-related quality of life
IBD: inflammatory bowel disease
IBDQ-9: IBD Questionnaire 9
ICT: information and communications technology
SCCAI: simple clinical colitis activity index
TECCU: Telemonitoring of Crohn Disease and Ulcerative Colitis
VAS: visual analog scale
5-ASA: 5-aminosalicylic acids
UC: ulcerative colitis
